# Fermented *Tamarindus indica* Seeds Mitigate Metabolic and Oxidative Stress in a *Nauphoeta cinerea* Model of STZ‐Induced Diabetes: Implications for Alzheimer’s disease

**DOI:** 10.1002/alz70859_106225

**Published:** 2025-12-25

**Authors:** Olayemi Philemon Aro, Opeyemi Babatunde Ogunsuyi, Ganiyu Oboh

**Affiliations:** ^1^ Universidade Federal do Rio Grande do Sul, Porto Alegre, Rio Grande do Sul Brazil; ^2^ Federal University of Technology, Akure, Ondo Nigeria; ^3^ Federal University of Technology Akure, Akure, Ondo Nigeria

## Abstract

**Background:**

Diabetes is a major risk factor for Alzheimer’s disease (AD), with shared pathological mechanisms including insulin resistance, oxidative stress, mitochondrial dysfunction, neuroinflammation, and impaired brain energy metabolism. Chronic hyperglycemia accelerates cognitive decline by promoting amyloid‐beta aggregation and tau hyperphosphorylation. Given the rising global prevalence of diabetes and its impact on neurodegeneration, plant‐based interventions with antioxidant and metabolic‐modulating properties hold promise for therapeutic development.

**Method:**

Cockroaches were divided into six groups: control, STZ‐induced, STZ+0.5% raw seeds, STZ+1% raw seeds, STZ+0.5% fermented seeds, and STZ+1% fermented seeds. Brain homogenates were analyzed for glucose, triglyceride, reactive oxygen species (ROS), and total thiol levels, biomarkers linked to metabolic and neurodegenerative disorders.

**Result:**

STZ‐induced cockroaches exhibited significant (p<0.05) elevations in glucose and ROS levels, along with a decline in total thiol content, indicative of oxidative damage. Treatment with tamarind seeds counteracted these effects, reducing ROS and glucose levels while increasing thiol content, suggesting enhanced antioxidant capacity. Survival rates were also improved in treated groups.

**Conclusion:**

Tamarind seed supplementation mitigated metabolic stress and oxidative damage, processes central to both diabetes and AD progression. These findings underscore the therapeutic potential of tamarind seeds in modulating key metabolic and neuroprotective pathways relevant to AD. Further research is needed to explore their impact on amyloid‐beta clearance, tau pathology, and neuroinflammatory responses in diabetic neurodegeneration models.

